# Improving the performance of models for one-step retrosynthesis through re-ranking

**DOI:** 10.1186/s13321-022-00594-8

**Published:** 2022-03-15

**Authors:** Min Htoo Lin, Zhengkai Tu, Connor W. Coley

**Affiliations:** 1 Division of Chemistry and Biological Chemistry, School of Physical and Mathematical Sciences, Nanyang Technological University, Singapore, 637371 Singapore; 2 Computational Science and Engineering, Massachusetts Institute of Technology, 77 Massachusetts Avenue, Cambridge, MA 02139 USA; 3 Department of Chemical Engineering, Massachusetts Institute of Technology, 77 Massachusetts Avenue, Cambridge, MA 02139 USA

**Keywords:** Computer-aided synthesis planning, Energy-based model, Cheminformatics, Machine learning, Synthetic chemistry

## Abstract

**Abstract:**

Retrosynthesis is at the core of organic chemistry. Recently, the rapid growth of artificial intelligence (AI) has spurred a variety of novel machine learning approaches for data-driven synthesis planning. These methods learn complex patterns from reaction databases in order to predict, for a given product, sets of reactants that can be used to synthesise that product. However, their performance as measured by the top-*N* accuracy in matching published reaction precedents still leaves room for improvement. This work aims to enhance these models by learning to re-rank their reactant predictions. Specifically, we design and train an energy-based model to re-rank, for each product, the published reaction as the top suggestion and the remaining reactant predictions as lower-ranked. We show that re-ranking can improve one-step models significantly using the standard USPTO-50k benchmark dataset, such as RetroSim, a similarity-based method, from 35.7 to 51.8% top-1 accuracy and NeuralSym, a deep learning method, from 45.7 to 51.3%, and also that re-ranking the union of two models’ suggestions can lead to better performance than either alone. However, the state-of-the-art top-1 accuracy is not improved by this method.

**Graphical Abstract:**

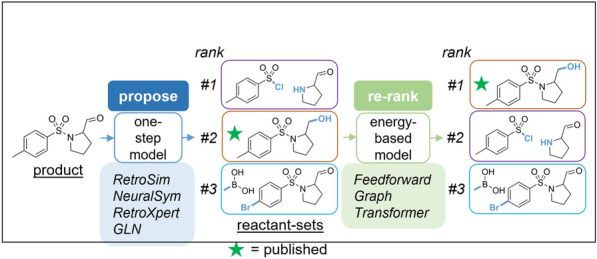

**Supplementary Information:**

The online version contains supplementary material available at 10.1186/s13321-022-00594-8.

## Introduction

At the core of organic chemistry, retrosynthesis is the process of deriving simpler precursors from a target molecule. Arguably first used by Robert Robinson in the synthesis of tropinone [1] and then formalized by Corey [2, 3], retrosynthesis decomposes a target into simpler reactants at each step, repeating this process recursively until we obtain commercially-available starting materials. However, retrosynthesis remains a challenging problem that can require years of experience and intuition to identify strategic disconnections for concise, practical routes. It can also be difficult to decide between multiple possible routes. At each step, chemists need to consider numerous factors from side reactions and ease of synthesis to reactant availability, not to mention the ever-expanding toolbox of reactions in the literature.

To help overcome these challenges, computer-aided synthesis planning (CASP) tools utilizing expert heuristics have been developed since the 1960s [2, 4] and have matured into high-performing, experimentally validated software [5]. Still, their reliance on hand-crafted rules requires much human effort and limits their ability to keep up with the fast-growing toolbox of reactions. In contrast, the growth of artificial intelligence as applied to chemistry has spurred novel data-driven approaches that learn complex patterns from reaction databases with much less human effort, and whose full potential we believe is yet to be realized. For examples of data-driven one-step retrosynthesis models, see NeuralSym [6], Seq2Seq [7], Retrosim [8], GLN [9], and MEGAN [10], all of which learn to predict a set of reactants given a product. As one-step models can be recursively applied to generate multi-step routes, here we focus on one-step retrosynthesis in line with these previous work.

Current data-driven methods can be broadly classified as template-based or template-free [11]. Template-based approaches, like NeuralSym [6], RetroSim [8] and GLN [9] use reaction “templates” [12] that specify the atoms and bonds at and around the reaction centre before and after the reaction. While chemically grounded, they require practitioners to strike a balance between the templates’ specificity and coverage, which is not trivial. To preserve generality across different molecules, algorithmically-extracted templates tend to focus on just reaction centres, losing information about distant functional groups which might cause unwanted reactions or be necessary for reactivity. Template-based methods also struggle to extrapolate to reactions beyond their template database [13]. In contrast, template-free methods learn the rules of retrosynthetic transformations implicitly from data without templates, aiming to achieve better generalization. Among these, sequence-based methods like Seq2Seq [7] and AutoSynRoute [14] treat retrosynthesis as a translation task from the language of products into the language of reactants, while graph-based methods like G2Gs [15] and MEGAN [10] perform graph-edits to convert the product graph into reactant graphs. Despite their competitive performance, it can be difficult to rationalise template-free predictions due to their black-box nature. As template-based models allow chemists to retrieve published precedents to explain the model’s predictions, they offer greater interpretability than template-free methods. All in all, there is value in both types of methods, depending on the use-case.

**Fig. 1:**
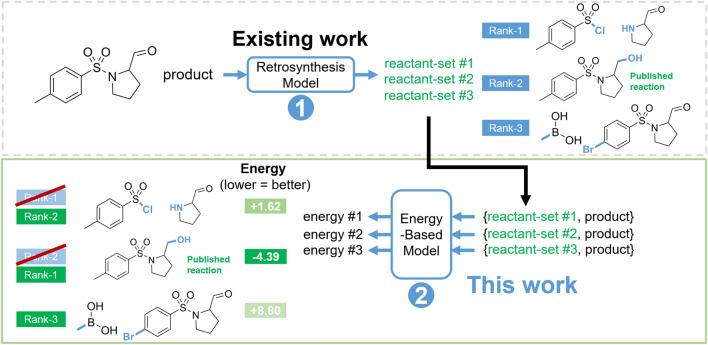
This work: re-ranking reactant suggestions proposed by existing one-step retrosynthesis models to recapitulate the published reaction

Typically, these data-driven methods are evaluated in terms of top-*N* accuracy, which measures whether the published reaction is present within the model’s top *N* suggestions. Rather than inventing another approach from scratch, we wondered if we could improve existing one-step models by learning to re-rank their predictions. Specifically, we were inspired by renewed interest in energy-based models (EBMs) [16, 17]. In our proposed formulation, the input to the EBM (Fig. 1) is a reaction (a product paired with a reactant-set suggested by a one-step model), with the EBM assigning an energy to each reaction. The term “energy” is a formalism and does not refer to physical energy. Instead, it can be seen as an inverse score (the lower the better) that holistically measures a reaction’s “feasibility”, implicitly learnt from data. This captures factors like reactivity, reactant availability, functional group compatibility, and how strategic the transformation is from a chemist’s perspective. For each product, the reactant-sets proposed are sorted from lowest to highest energy; the reactant-set assigned the lowest energy by the EBM is the EBM’s top re-ranked prediction. Therefore, a trained EBM can be used for a re-ranking step after each one-step prediction to improve the one-step model’s ability to recapitulate the “true” retrosynthetic strategies in the literature.

It should be noted that re-ranking and verifying suggestions for forward predictions and retrosynthesis have been studied in the past. Initial work done by Satoh and Funatsu [18] evaluates forward reactions using a rule-based approach with pattern matching. More recently, Segler et al. implemented an in-scope filter [19] by training a model on a simple binary classification task of whether a reaction is feasible. In this case, “positive” (feasible) reactions were contained in reaction databases, while “negative” (infeasible) reactions were artifcially generated through the use of forward reaction templates. Schwaller et al. [20] alternatively used a transformed-based forward prediction model [21] to score retrosynthetic suggestions in terms of their likelihood of producing the intended product, which was used in combination with a modified version of the SCScore [22] to prioritize steps in the RXN synthesis planning tool [23]. Our energy-based re-ranking approach inherently differs from these prior work as we use hard negatives sampled from trained one-step proposers.

Additionally, Sun et al. [24] recently applied the EBM framework to retrosynthesis. Their main contributions include formulating an energy-based view of retrosynthesis, showing theoretically that both template-based and template-free models can be re-framed as EBMs that output energy values. They also applied the concept of re-ranking with a novel dual Transformer model to assign energies and re-rank reactant-sets proposed by another Transformer model, which did improve upon the proposing Transformer model’s performance, from an original 44.4% to 53.6% re-ranked top-1 accuracy. This dual Transformer considers not only the backward reaction direction (retrosynthesis), but also the forward direction, and learns to keep both directions in agreement. To the best of our knowledge, while they did make use of generated negatives to train this dual variant, it was not for the purpose of explicitly training re-rankers like we do. Explicitly training the re-ranker on proposals from the proposing model might teach the re-ranker useful insights about what differentiates a good reactant proposal from a bad one. In addition, we explore a wider range of proposer and re-ranker combinations beyond just Transformer-based ones. Ultimately, their work positively suggests the potential of energy-based approaches to re-ranking and serves as inspiration for our work.

## Methods

### The energy-based re-ranking model

Motivated by statistical mechanics, EBMs describe the unnormalized probability distribution $p_\theta$ of a variable of interest, *x*, using an energy function $E_\theta (x) : {\mathbb {R}}^D \mapsto {\mathbb {R}}$. Popularized in mid-2000s [25], EBMs regained interest after recent works drawing theoretical connections between EBMs and the widespread tasks of classification and generation [16, 17]. Formally, we have:

1 $$\begin{aligned} p_\theta (x) = \frac{e^{-E_\theta (x)}}{Z(\theta )}\qquad Z(\theta ) = \int _xe^{-E_\theta (x)}dx \end{aligned}$$

EBMs are highly flexible as there is no restriction to the form of the energy function $E_\theta$, allowing the user massive freedom to explore and utilize what best suits the problem at hand. We exploit this flexibility by exploring different architectures of deep neural networks that encode different molecular representations to parameterize $E_\theta$. The ranking architecture choice for the ranking model can be chosen independently from the proposer model. Given an input reaction *x*, the output of our network, $E_\theta (x)$, is its “energy”, a scalar value (the lower the better), which holistically represents that reaction’s feasibility. Also, notice that the denominator $Z(\theta )$ in Eq. 1, the partition function, requires integrating $E_\theta$ over all possible input reactions in order to represent a meaningful probability distribution. However, this partition function is computationally intractable and it is necessary to simplify it. In the context of retrosynthesis, for each product, a one-step model can generate up to *K* reactant-sets: $\left\{ R_k \right\} _{k=1}^K$. While only one of these can exactly match the published reactant-set (“ground-truth”, $R_{true}\in \left\{ R_k \right\} _{k=1}^K$), given a well-trained one-step model, several of the remaining reactant-sets, $\left\{ R_k \right\} _{k=1}^K\setminus R_{true}$, could also be chemically viable options or could be similar to $R_{true}$ (differing by the identity of a leaving group, for example). Therefore, we reasoned that we could simply use these remaining reactant-sets as “negatives” to approximate the intractable partition function $Z(\theta )$. Similar simplification was also made in Sun et al’s energy-based modelling work [24] which shares our theoretical motivations but samples the “negatives” in a different way. They choose to assume that extracted reaction template sets are exhaustive and subsequently apply non ground-truth templates to generate negative samples, while we assume that relevant proposed reactants are exhaustive. We then empirically show in this work that such an approximation is sufficient for good retrosynthesis re-ranking performance.

Therefore, the EBM’s training objective is to describe the dataset of reactions to maximize the separation of energy between a positive reaction (a product paired with published reactant-set) against its associated negatives (the same product paired with non-published reactant-sets proposed by the one-step model), by pushing down the energy of positive reactions while pushing up the energy of negative reactions. To achieve this, we design the following loss function, where for a product *P*, given a batch of top *K* reactant-set proposals $\left\{ R_k \right\} _{k=1}^K$ from a one-step model, we have:

2 $$\begin{aligned} {\mathcal {L}}_{batch} = -\log \,p_\theta \left( R_{true},\, \{R_k\} ,\,P\right) = -\log \,\left( \frac{e^{-E_\theta (R_{true}, \,P)}}{\sum _{k=1}^{K}e^{-E_\theta (R_k,\,P)}}\right) \end{aligned}$$

The EBM can then be trained using this loss function through stochastic gradient descent and variants over mini-batches of data. This approach has conceptual similarities to contrastive learning, where the decision to use negative examples from existing one-step models can be thought of as a form of hard negative sampling. Theoretically, the higher the *K*, the better the approximation to the true partition function $Z(\theta )$, but in practice, we do not find any noticeable benefit in using $K > 50$, and therefore use $K = 50$ for all experiments. We also note that because one-step models do not have a perfect top-50 accuracy, the set of top *K* proposals will not always contain the published reactant-set. During training, we add the ground truth reactant-set $R_{true}$ to the list of candidates if it is not already present. When re-ranking validation or test reactions, however, if $R_{true}$ is not part of the top *K* proposals from the one-step model given a product, the EBM cannot re-rank correctly for this product. To maximize the chances of $R_{true}$ being present in $\left\{ R_k \right\} _{k=1}^K$, we use a larger $K = 200$ when re-ranking validation and test proposals.

### Architecture choices for the energy-based model

Due to the flexibility of the EBM framework, we enjoy great freedom in both input representation and architecture. In this work, we focus on two machine-readable formats to represent a chemical reaction, with each choice corresponding to a different architecture below. We explore both a feedforward backbone and a graph-based Message Passing Neural Network (MPNN) backbone. We did briefly experiment with a Transformer-based architecture, but did not observe good performance and further discuss it in Additional file 1: Section S4.3.3. For all three architectures, we elaborate on details of the network structure and hyperparameter choice in the Additional file 1: Section S4.

#### Feedforward EBM (FF-EBM)

**Fig. 2:**
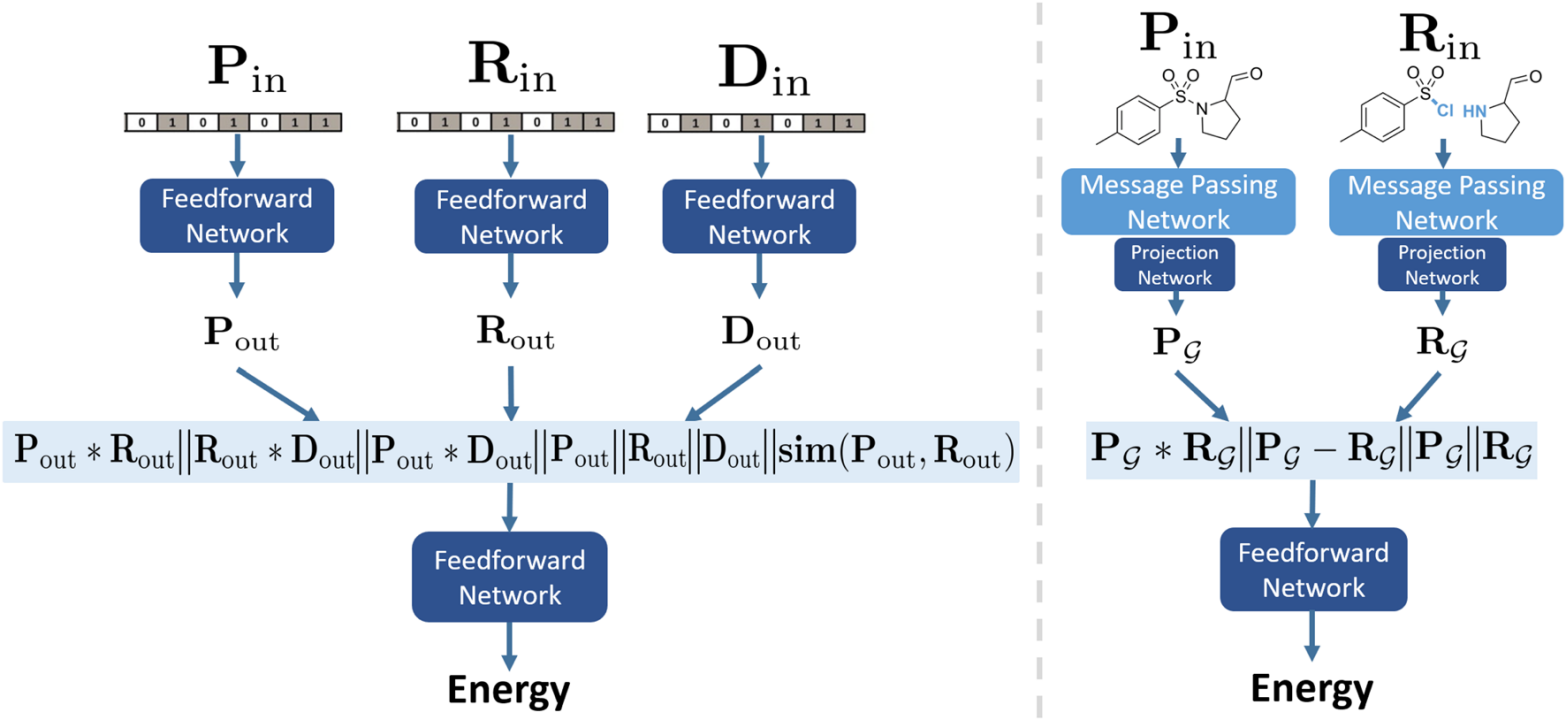
FF-EBM (left) and Graph-EBM (right) schematics; $\mathbin \Vert$ stands for concatenation

We represent each molecule as a Morgan count fingerprint of length 16,384 with radius 3, and employ 3 input networks (Fig. 2, left). The first network receives the product fingerprint $\mathbf{P }_\text {in}$. The second network receives the reactants fingerprint $\mathbf{R }_\text {in}$; because each reaction can have multiple reactants, we sum reactant fingerprints into a single, 16,384-length fingerprint. Lastly, the third network receives the “difference” fingerprint [26] $\mathbf{D }_\text {in}$, which captures fragments lost and gained during the reaction: $\mathbf{D }_\text {in} = \mathbf{P }_\text {in} - \mathbf{R }_\text {in}$. From these 3 input networks, we obtain 3 dense embeddings $\mathbf{P }_\text {out}$, $\mathbf{R }_\text {out}$, $\mathbf{D }_\text {out}$. We concatenate these 3 vectors with their element-wise products $\mathbf{P }_\text {out} *\mathbf{R }_\text {out}$, $\mathbf{R }_\text {out} * \mathbf{D }_\text {out}$ and $\mathbf{P }_\text {out} * \mathbf{D }_\text {out}$ to capture higher-order interactions as inspired by Ref. [27], as well as the cosine similarity of product and reactants embeddings $\mathbf{sim} (\mathbf{P }_\text {out},\mathbf{R }_\text {out}) = \frac{\mathbf{P }_\text {out}\cdot \mathbf{R }_\text {out}}{\left\| \mathbf{P }_\text {out} \right\| \left\| \mathbf{R }_\text {out} \right\| }$. Finally, we apply another feedforward network on this concatenated vector to output the energy.

#### Graph EBM

A graph ${\mathcal {G}}$ contains nodes ${\mathcal {V}}$ corresponding to atoms and edges ${\mathcal {E}}$ that link nodes, corresponding to bonds. We adapt the graph representation from GraphRetro [28]. Each atom *u* has a feature vector $\mathbf{x }_u$ containing chemical properties such as its element and charge. Similarly, each bond (*u*, *v*) between two atoms *u* and *v* has a feature vector $\mathbf{x }_{uv}$, containing information like bond type. The full list is detailed in Additional file 1: Table S1. We directly adapt the graph encoder from Ref. [28]. The MPNN performs a defined number of message passing operations around each atom and bond in the molecule, which communicates chemical information from neighbouring bonds and atoms to extract meaningful representations. These representations can be very powerful as they are custom-learnt for the task at hand. In contrast, fingerprints are constructed using a fixed algorithm agnostic to the task, which may not yield optimal performance. For brevity, we denote the MPNN’s encoding process by MPNN($\cdot$) and refer readers to Refs. [28, 29] for detailed explanations of the MPNN architecture. In our case, we apply two MPNNs with separate parameters (Fig. 2, right): one MPNN just for the reactant graphs, and the other just for the product graph. For each graph ${\mathcal {G}}$, the MPNN computes atom representations $\mathbf{c }_u$ for each atom *u*, i.e. $\left\{ \mathbf{c }_u|u\in {\mathcal {G}} \right\}$, using Eq. 3:

3 $$\begin{aligned} \left\{ \mathbf{c }_u \right\} = MPNN ({\mathcal {G}}, \left\{ \mathbf{x }_u \right\} , \left\{ \mathbf{x }_{uv} \right\} _{v\in {\mathcal {N}}(u)}) \end{aligned}$$

where ${\mathcal {N}}(u)$ refers to the neighbouring atoms of atom *u*. To obtain a fixed-length graph-level embedding for each molecule $\mathbf{c }_{\mathcal {G}}$, all of its atom representations could be summed: $\mathbf{c }_{\mathcal {G}} = \sum _{u\in {\mathcal {V}}}\mathbf{c }_u$. However, a naive sum may not be optimal as certain atoms may be more important in determining a reaction’s feasibility. Thus, we use attention pooling [30] which uses a feedforward network to calculate the weight (“attention”) each atom should contribute to the graph-level embedding in a weighted sum. Since a reaction can have multiple reactants, we sum the graph-level embeddings of all reactants into a single vector. We additionally apply a small projection network to the pooled output from each MPNN encoder to respectively obtain the product embedding $\mathbf{P }_{{\mathcal {G}}}$ and reactants embedding $\mathbf{R }_{{\mathcal {G}}}$. Finally, we concatenate $\mathbf{P }_{{\mathcal {G}}}$ and $\mathbf{R }_{{\mathcal {G}}}$ with their difference $\mathbf{P }_{{\mathcal {G}}} - \mathbf{R }_{{\mathcal {G}}}$ and element-wise product $\mathbf{P }_{{\mathcal {G}}} * \mathbf{R }_{{\mathcal {G}}}$, before applying an output feedforward network to obtain the energy.

### Dataset and preprocessing

We trained our models on the USPTO-50K dataset of roughly 50,000 reactions extracted from the United States patent literature from 1976 to 2016 [31]. The reactions are recorded as atom-mapped SMILES strings and comprise 10 types (Additional file 1: Table S2). We use a cleaned version of the random 80%/10%/10% split from Refs. [8, 9], where additional duplicate reactions are removed (explained in Additional file 1: Section S3), for a total of 39,713 training, 4989 validation and 5005 test reactions.

### One-step models used for re-ranking

We re-trained from scratch all the one-step models to be re-ranked—RetroSim [8], NeuralSym [6], GLN [9] and RetroXpert [32]—on our cleaner USPTO-50K, which led to minor discrepancies in quantitative top-*N* accuracies relative to previously reported values. However, results are typically within 2% of previously reported values. RetroSim [8] is a template-based approach that computes and compares molecular similarity to choose the best template for a given product. The similarity is a combination of product similarity and reactants similarity, calculated against the training data. Using only similarity values, RetroSim is a purely data-driven method without any model parameters. Next, NeuralSym [6] is a one-step retrosynthesis deep-learning model trained to classify, for a given product in each retrosynthesis step, the most relevant template from a library of templates algorithmically-extracted from the training set. One of the best performing template-based models, the Graph Logic Network (GLN) [9] is parameterized by graph neural networks. First, GLN identifies reaction centres in a given product, and then ranks the most relevant templates, before finally scoring and matching reactants given each template. In the realm of template-free methods, Retrosynthesis eXpert (RetroXpert) [32] is a hybrid graph-sequence model that employs a graph network to first identify likely reaction centres to decompose the product into synthons, followed by a Transformer network to generate full reactants from each synthon.

### Implementation

We used several open-source libraries with Python 3.6 [33]. PyTorch [34] was used as the backbone for building, training and testing all models. We used RDKit [35] and rdchiral [36] for chemical processing, and NetworkX [37] for processing molecular graphs for the Graph-EBM. As NeuralSym is not open-sourced, we re-implemented it from scratch at https://github.com/linminhtoo/neuralsym following the original work [6]. All code for data preprocessing, proposer training, EBM training and evaluation is open-sourced at: https://github.com/coleygroup/rxn-ebm.

We tuned the hyperparameters for each of the three EBM architecture by choosing the hyperparameters that produce the best top-1 accuracy on the validation data. It is also possible to optimize for other top-*N* accuracies, if desired. The tuning was done manually starting from common settings found in the literature. Only after the best hyperparameters for each EBM architecture have been finalized, we then calculate and report the top-*N* accuracies with these optimized hyperparameters on the USPTO-50K test data for re-ranking each of the four one-step models. Specific model and training hyperparameters are described in Additional file 1: Section S4.

## Results

### Re-ranking individual models

The first one-step model, RetroSim, has been greatly improved by Graph-EBM, from 35.7% to 51.8% top-1 accuracy (a relative factor of 45%) as shown in Table 1. The remaining top-*N* accuracies for $N\in \left\{ 3, 5, 10, 20\right\}$ are also significantly boosted. The Graph-EBM is clearly superior than the FF-EBM, although not by a very large margin.

**Table 1:** Results of re-ranking four one-step models on the USPTO-50K test dataset

Models	Top-*N* accuracy (%)						Mean Reciprocal Rank
	1	3	5	10	20	50	
RetroSim	35.7 ($\pm 0$)	53.3 ($\pm 0$)	62.0 ($\pm 0$)	73.4 ($\pm 0$)	82.3 ($\pm 0$)	88.5 ($\pm 0$)	0.477 ($\pm 0.000$)
RetroSim + FF-EBM	49.7 ($\pm 0.34$)	72.3 ($\pm 0.21$)	79.4 ($\pm 0.15$)	85.5 ($\pm 0.13$)	88.1 ($\pm 0.07$)	**88.9** ($\pm 0.01$)	0.622 ($\pm 0.002$)
RetroSim + Graph-EBM	**51.8** ($\pm 0.43$)	**74.5** ($\pm 0.37$)	**81.1** ($\pm 0.17$)	**86.4** ($\pm 0.13$)	**88.5** ($\pm 0.02$)	**88.9** ($\pm 0.00$)	**0.644** ($\pm 0.004$)
NeuralSym	45.7 ($\pm 0.30$)	66.4 ($\pm 0.40$)	73.5 ($\pm 0.30$)	80.7 ($\pm 0.21$)	85.3 ($\pm 0.34$)	87.3 ($\pm 0.32$)	0.578 ($\pm 0.001$)
NeuralSym + FF-EBM	50.5 ($\pm 0.21$)	71.8 ($\pm 0.62$)	78.7 ($\pm 0.18$)	84.5 ($\pm 0.32$)	87.1 ($\pm 0.29$)	**87.5** ($\pm 0.32$)	0.626 ($\pm 0.003$)
NeuralSym + Graph-EBM	**51.3** ($\pm 0.52$)	**73.6** ($\pm 0.34$)	**80.2** ($\pm 0.35$)	**85.4** ($\pm 0.30$)	**87.1** ($\pm 0.27$)	**87.5** ($\pm 0.32$)	**0.636** ($\pm 0.004$)
RetroXpert	**45.8** ($\pm 0.25$)	59.2 ($\pm 0.26$)	63.0 ($\pm 0.57$)	66.9 ($\pm 0.31$)	69.9 ($\pm 0.62$)	73.0 ($\pm 0.70$)	**0.543** ($\pm 0.004$)
RetroXpert + FF-EBM	42.7 ($\pm 0.27$)	**62.0** ($\pm 0.21$)	**67.6** ($\pm 0.05$)	72.5 ($\pm 0.08$)	75.6 ($\pm 0.11$)	77.1 ($\pm 0.20$)	0.536 ($\pm 0.002$)
RetroXpert + Graph-EBM	36.7 ($\pm 0.91$)	58.2 ($\pm 1.06$)	65.8 ($\pm 0.73$)	**73.0** ($\pm 0.32$)	**75.9** ($\pm 0.12$)	**77.3** ($\pm 0.21$)	0.491 ($\pm 0.008$)
GLN	51.7 ($\pm 0.33$)	67.8 ($\pm 0.43$)	75.1 ($\pm 0.32$)	83.2 ($\pm 0.12$)	88.9 ($\pm 0.11$)	92.4 ($\pm 0.06$)	0.620 ($\pm 0.003$)
GLN + FF-EBM	49.7 ($\pm 0.77$)	72.4 ($\pm 0.18$)	80.0 ($\pm 0.28$)	87.0 ($\pm 0.11$)	90.6 ($\pm 0.12$)	**93.0** ($\pm 0.02$)	0.629 ($\pm 0.005$)
GLN + Graph-EBM	**52.3** ($\pm 0.01$)	**74.9** ($\pm 0.27$)	**82.0** ($\pm 0.18$)	**88.0** ($\pm 0.02$)	**91.4** ($\pm 0.11$)	**93.0** ($\pm 0.08$)	**0.652** ($\pm 0.001$)

Bolded values refer to the best top-*N* accuracy and the best MRR for that one-step model. We report the average of 3 experiments where both the proposer and re-ranker are initialized with a different random seed, with the standard deviation in parentheses. Note that RetroSim is a deterministic algorithm and is reported with a standard deviation of 0

NeuralSym’s performance is also significantly enhanced by both FF-EBM and Graph-EBM, although the margin is not as large as for RetroSim. The Graph-EBM is again slightly superior than FF-EBM across the board, particularly for $N\in \left\{ 3,5\right\}$. RetroXpert, however, paints a different picture. Firstly, both FF-EBM and Graph-EBM fail to recover RetroXpert’s original top-1 accuracy, much less improve it. More surprisingly, the Graph-EBM suffers a large drop in top-1 accuracy from RetroXpert’s 45.8% to 36.7%, which is noticeably worse than the FF-EBM’s 42.7%. On the positive side, the FF-EBM does improve upon RetroXpert’s top-3 to top-50 accuracies, while the Graph-EBM does so for top-5 onwards; the FF-EBM is superior to the Graph-EBM up to $N=5$, beyond which the Graph-EBM takes over.

Lastly, for GLN, the best one-step model among these four in terms of top-1 accuracy, only the Graph-EBM is able to improve (albeit marginally) the top-1 accuracy from 51.7% to 52.3% while the FF-EBM falls short at 49.7%. Still, we observe solid enhancements of the top-3 to top-50 accuracies. On the whole, the general improvement in top-*N* accuracies by both our FF-EBM and Graph-EBM highlight that our energy-based re-ranking approach can indeed improve the performance of a range of one-step models, *relative* to their original performance.

In addition to top-*N* accuracy, we also compare the one-step models’ performance before and after re-ranking using two other metrics, the Mean Reciprocal Rank (MRR), which is also shown in Table 1, as well as area under the top-*N* curve (Additional file 1: Section S5.1). With MRR, just as with top-*N* accuracy, re-ranking with Graph-EBM achieves the best performance on RetroSim, NeuralSym and GLN, compared to re-ranking with FF-EBM. For RetroXpert, re-ranking with FF-EBM gives better MRR than the Graph-EBM, but both re-ranked versions are worse than the original RetroXpert. On the whole, the trends in MRR mirror the same patterns seen with top-*N* accuracy.

### Re-ranking multiple models

**Fig. 3:**
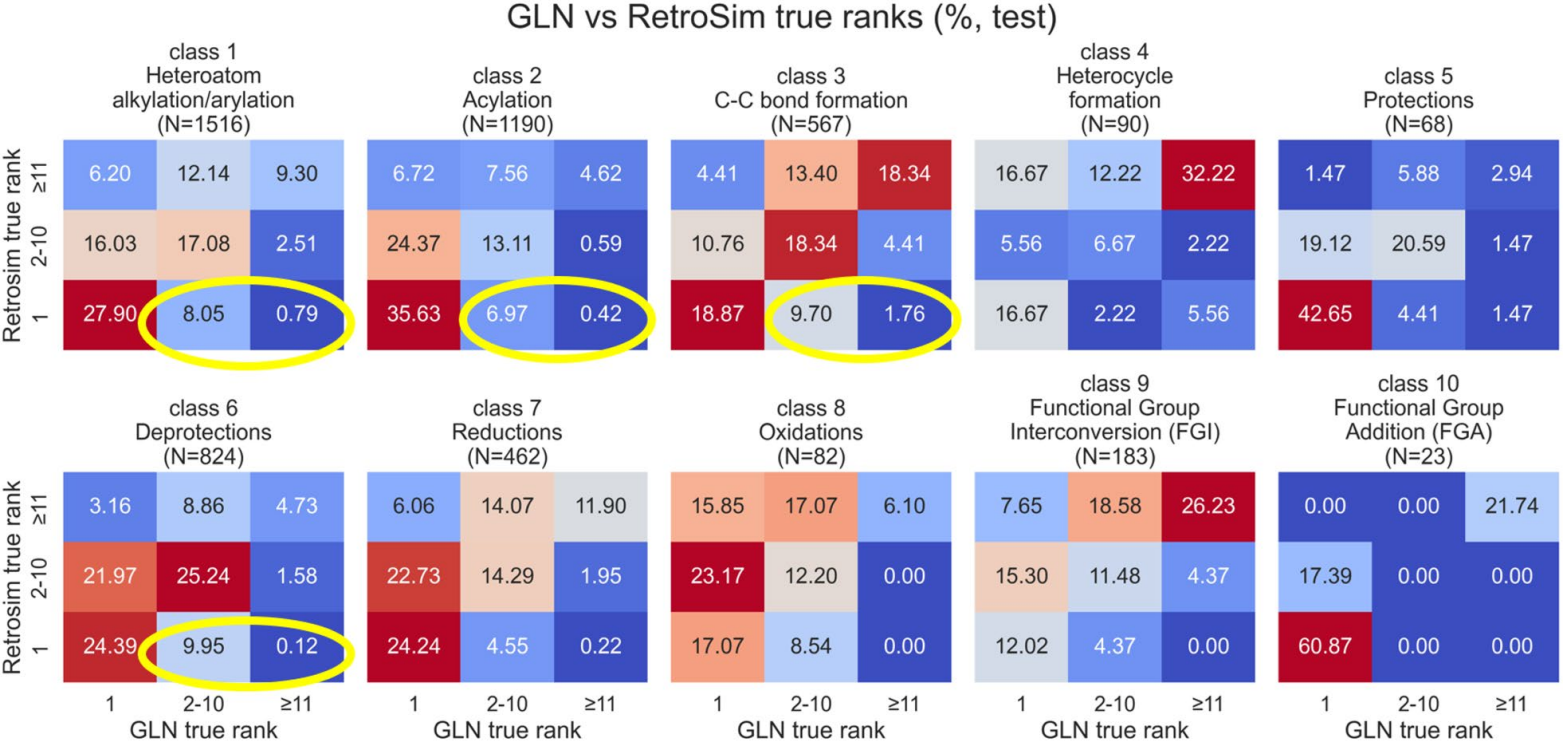
Comparing GLN vs RetroSim across the 10 reaction classes. Note that figures are taken from one specific seed of each model and not averaged over 3 replicates

As each retrosynthesis model is inherently different in design, we conducted a detailed analysis of the correlation between proposals from different models. Indeed, we found that each model does get a different subset of reactions correct. In Fig. 3, we collate and compare the frequency of different ranks assigned by GLN and RetroSim to each published reactant-set in the USPTO-50K test data. As an example, if GLN’s true rank is 2 for a product, it means that GLN’s rank-2 proposal matches the published reactant-set.

Across almost every class but particularly in classes 1, 2, 3 and 6 (circled in yellow; classes defined in Additional file 1: Table S2), we observe between 7 and 10% of reactions where RetroSim scored the published reactants as its rank-1 prediction, but GLN only got them as its rank-2 (or worse) prediction. Thus, even though GLN is significantly superior to RetroSim *on average*, with a top-1 accuracy of 51.7% versus 35.7%, RetroSim does better on subsets of reactions. We note similar trends when comparing other models, such as GLN against RetroXpert. In short, this analysis encouraged us to check if the EBM could learn to re-rank proposals combined from multiple models. If it can learn to leverage the “strong chemical space” from each model, we could hope for an even larger margin of improvement than re-ranking each model individually. To investigate this, we trained a Graph-EBM on proposals pooled from RetroSim and GLN, with up to 50 from each model during training, and 200 each during testing. Therefore, the Graph-EBM sees up to 100 proposals during training and up to 400 during testing, as opposed to just 50 and 200 when re-ranking models individually.

**Table 2:** Results of re-ranking combined proposals of GLN and RetroSim on USPTO-50K test data

Models	Top-*N* accuracy (%)						Mean Reciprocal Rank
	1	3	5	10	20	50	
RetroSim	35.7 ($\pm 0$)	53.3 ($\pm 0$)	62.0 ($\pm 0$)	73.4 ($\pm 0$)	82.3 ($\pm 0$)	88.5 ($\pm 0$)	0.477 ($\pm 0.000$)
RetroSim + Graph-EBM	51.8 ($\pm 0.43$)	74.5 ($\pm 0.37$)	81.1 ($\pm 0.17$)	86.4 ($\pm 0.13$)	88.5 ($\pm 0.02$)	88.9 ($\pm 0.00$)	**0.644** ($\pm 0.004$)
GLN	51.7 ($\pm 0.33$)	67.8 ($\pm 0.43$)	75.1 ($\pm 0.32$)	83.2 ($\pm 0.12$)	88.9 ($\pm 0.11$)	92.4 ($\pm 0.06$)	0.620 ($\pm 0.003$)
GLN + Graph-EBM	52.3 ($\pm 0.01$)	74.9 ($\pm 0.27$)	82.0 ($\pm 0.18$)	88.0 ($\pm 0.02$)	91.4 ($\pm 0.11$)	93.0 ($\pm 0.08$)	**0.652** ($\pm 0.001$)
GLN + RetroSim + Graph-EBM	**52.5** ($\pm 0.10$)	**75.7** ($\pm 0.15$)	**83.1** ($\pm 0.34$)	**89.7** ($\pm 0.18$)	**93.1** ($\pm 0.12$)	**94.8** ($\pm 0.06$)	**0.658** ($\pm 0.000$)

Bolded values represent best top-*N* accuracies and best MRR across both GLN and RetroSim (including their individually re-ranked versions)

Positively, all of the top-*N* accuracies as well as MRR have been further improved (Table 2) beyond re-ranking just GLN with the Graph-EBM, with the largest margins of improvement in top-*N* accuracy for $N \in \left\{ 10, 20, 50\right\}$. This indicates that the EBM does benefit from seeing a wider variety of proposals and is able to learn to utilize the unique strengths of RetroSim and GLN. It may help to supplement each proposal with the original probability or rank assigned by each one-step model. We did experiment re-ranking more than 2 one-step models, but this did not yield better top-*N* accuracies than re-ranking 1 or 2 one-step models, and did not pursue it further.

## Discussion

### Training proposals vs test proposals

We note that the EBM re-ranking performance on RetroXpert is poor compared to the other 3 one-step models, especially in terms of top-1 and top-3 accuracies. Although the exact reasons for this remain unclear to us, one hypothesis is that there is significant divergence between the distribution of RetroXpert’s training proposals versus its test proposals. That is, RetroXpert’s incorrect predictions on the training set (to which it has been (over)fit) will be mistakes of a different kind compared to incorrect predictions on the test set. This makes it more challenging for our EBMs to generalise the patterns they have learnt about RetroXpert’s training proposals to its test proposals. One possible solution might be to set aside a larger validation dataset when training RetroXpert. Subsequently, we can use RetroXpert’s proposals on that validation dataset to train the EBM re-ranker, rather than its proposals on the training dataset. Since the validation and test proposals should be very similar in distribution, such a set-up could improve the EBMs’ generalisation. However, a trade-off is that we have less data to train the one-step model, which is likely to worsen its performance. It remains to be investigated whether these two conflicting factors would overall boost or worsen the combined proposal-re-ranking performance.

### Limitations of top-*N* accuracy

For a reaction to be top-1 accurate, the published reactants has to exactly match the one-step model’s highest-ranked suggestion but there almost always exist multiple plausible routes to a product. Hence, teaching and validating models to recapitulate the published reaction may not be ideal, since it is not necessarily the “best” reaction (a highly subjective concept). We acknowledge that top-*N* accuracy alone does not paint a complete picture of a one-step model’s performance, as others have also argued [20]. Unfortunately, chemical plausibility is subjective without specification of reaction conditions and, moreover, is difficult to verify without time-consuming experiments; the use of reaction prediction models for evaluation is not always ideal as these models come with their own errors and could complicate analysis, although they can be useful in injecting prior chemical biases. Therefore, top-*N* accuracy continues to be a primary metric in computer-aided retrosynthesis as it is quantitative, scalable and enables standardised comparison of models across benchmark datasets.

### Multi-step synthesis planning

Our energy-based re-ranking model can be applied to multi-step synthesis planning by using it to re-rank each single step during a recursive expansion. In general, the re-ranking stage merely reorders proposed precursors at each single step. However, this may complicate existing pipelines that rely on precursor scores, such as [19]’s Monte Carlo Tree Search which directly uses probabilities assigned by the NeuralSym policy network. In addition, the energy score is compatible with several existing scores used to shortlist single-step retrosynthetic suggestions, such as a forward prediction score [21] or a synthesizability score [22]. One simple approach could be to linearly combine multiple single-step scores by tuning the scalar coefficients on a validation set, and use the combined scores for re-ranking at each step. As the focus of this work is on single-step re-ranking, we leave both of these considerations to future work.

### Visualizing re-ranked predictions

We supplement our quantitative evaluations with qualitative case studies to highlight the plausibility of our EBM’s re-ranked predictions. Prior works [8, 9, 32] have also utilised similar visualisations to support their case and analyse their one-step models, but we acknowledge that a small number of examples does not provide a comprehensive view of what has been learned. In Additional file 1: Section S6, we also display examples where re-ranking has egregiously worsened the rank of the published reaction.

**Fig. 4:**
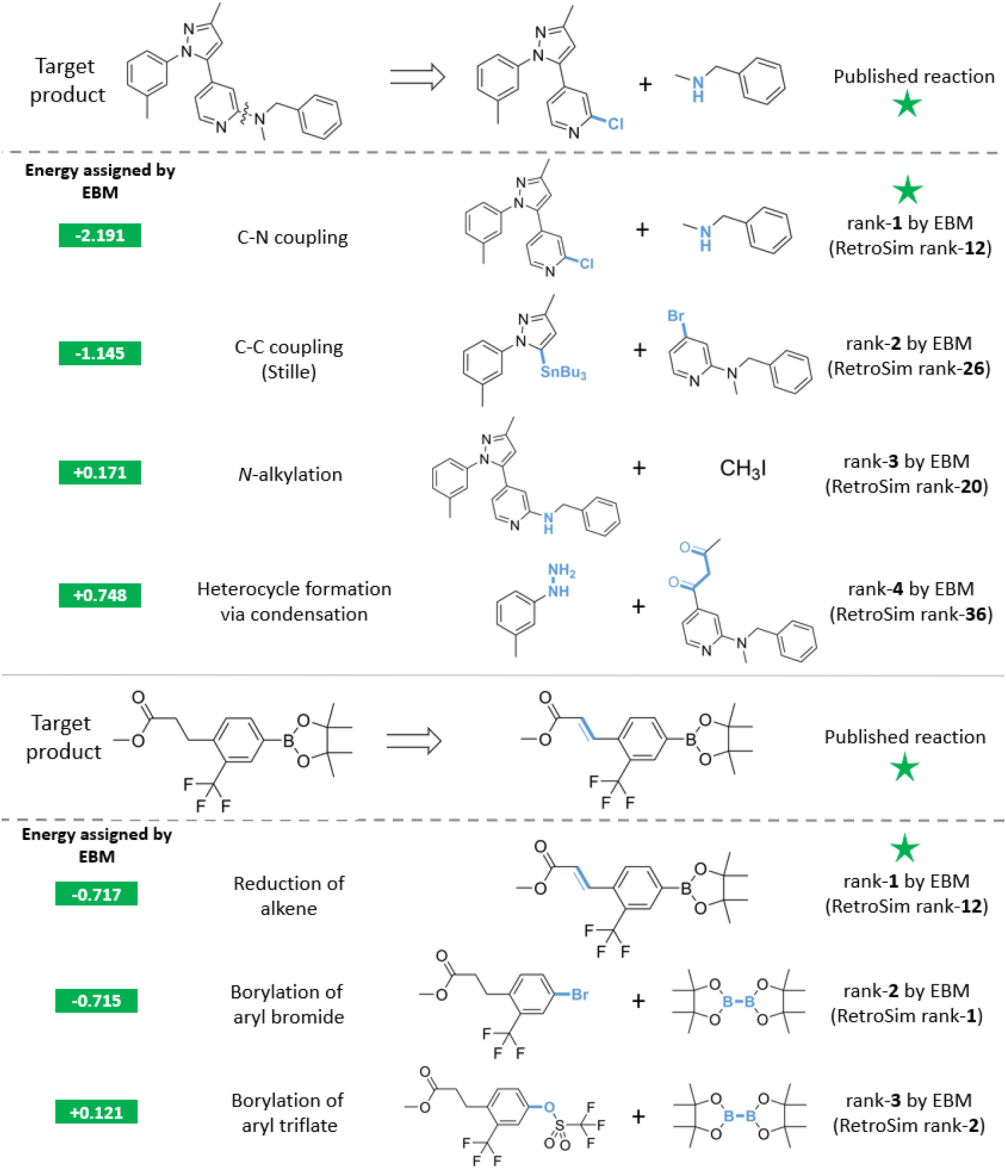
Top: our EBM re-ranks the published reaction correctly and the EBM’s other top suggestions are also chemically reasonable despite being poorly ranked by RetroSim; bottom: another successful re-ranking by the EBM, with rank-2 and rank-3 suggestions also plausible reactions

#### Re-ranked reactants that match published reactants

First, we highlight examples of proposals that were successfully re-ranked, where the one-step model’s original rank-1 prediction did not match the published reactants. We focus on examples from re-ranking RetroSim with the Graph-EBM, as this effected the greatest margin of improvement. In the first example (Fig. 4, top), the published reaction is a C–N coupling between a secondary alkyl amine and a 2-chloropyridine. Although the published reaction is not favored by RetroSim with a rank of 12, the Graph-EBM recognizes its feasibility and assigned it the lowest energy. Furthermore, the EBM recovers promising reactions ranked poorly by RetroSim. The top-2 suggestion by the EBM is a Stille reaction between an organostannane and an aryl bromide, a valid route that is not favored by RetroSim with an original rank of 26. The EBM’s rank-3 suggestion is an *N*-alkylation of the secondary aryl amine with methyl iodide, which is chemically plausible, but ranked 20th by RetroSim. Similarly, EBM’s rank-4 suggestion is a condensation between a hydrazine moiety and a diketone to construct the pyrazole ring. This, too, is a known, real reaction but only ranked 36th by RetroSim.

Next in Fig. 4 (bottom), the published reaction involves reducing a conjugated alkene to an alkane. Although RetroSim ranked this reaction only 12th, our EBM again re-ranked it as its top suggestion. The EBM’s rank-2 and rank-3 predictions are the borylation of aryl bromide and aryl triflate respectively, which are both realistic suggestions and this time also ranked highly by RetroSim as rank-1 and rank-2.

**Fig. 5:**
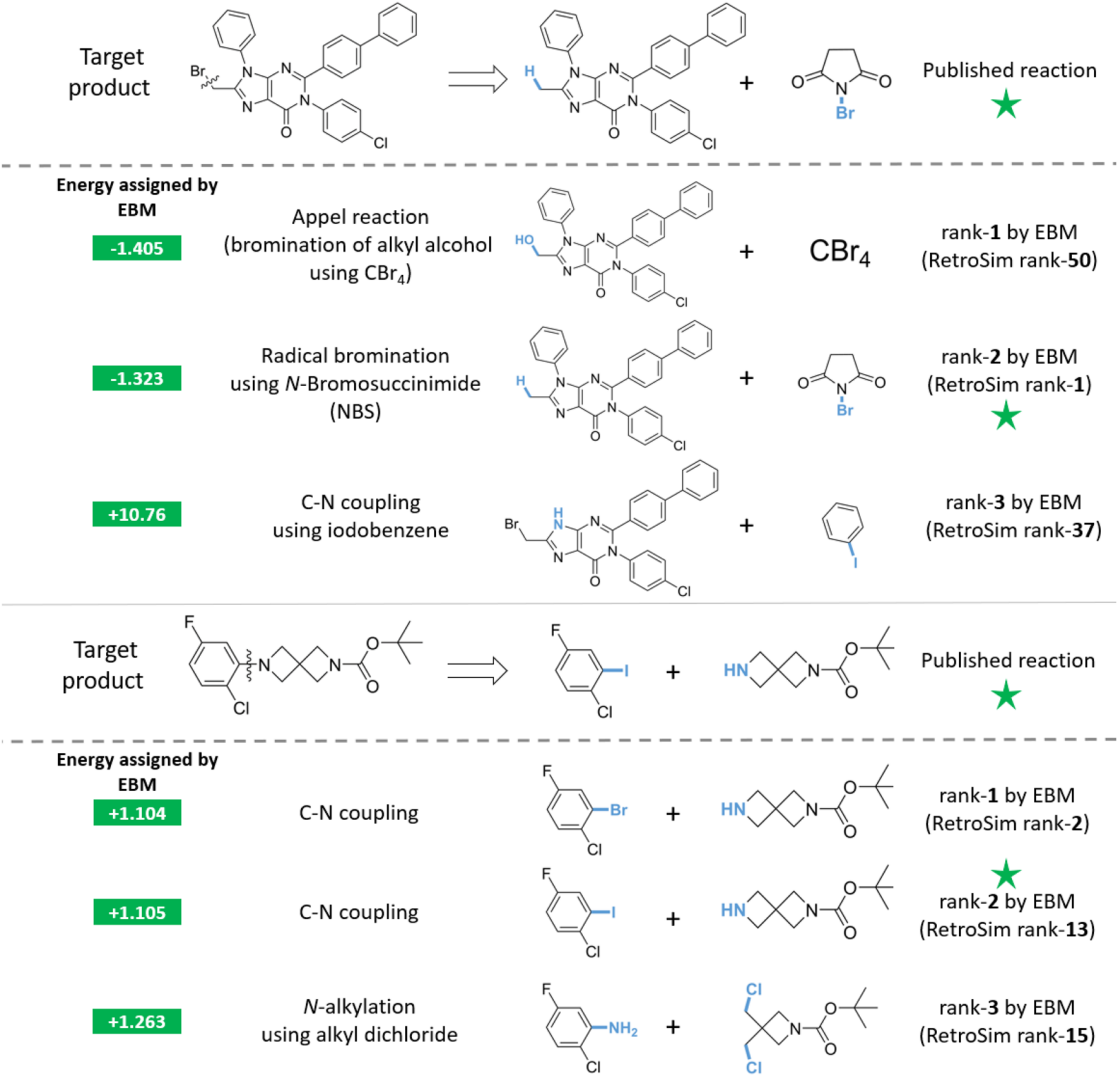
Top and bottom: two examples of the EBM’s rank-1 suggestion being reasonable despite not matching the published reaction

#### Re-ranked reactants that do not match published reactants

We also analyse cases where the EBM failed to re-rank the published reactants as rank-1. In Fig. 5 (top), the published reaction is the radical bromination of a methyl arene group using *N*-Bromosuccinimide. The EBM’s rank-1 prediction is different: it slightly prefers the bromination of an alkyl alcohol using tetrabromomethane. This is, in fact, the well-known Appel reaction [38] which is certainly a plausible choice. Multiple reasonable routes often exist, and our EBM’s suggestions are still valuable even when they differ from the published reaction. On the other hand, the EBM’s rank-3 proposal is a C–N coupling between iodobenzene and the NH nitrogen of the purine core. This suggestion seems slightly less favorable due to potential side reactions such as nucleophilic attack at the alkyl bromide or aryl chloride groups. What is remarkable is that the EBM assigns a significantly higher energy to this (+10.76) than its rank-1 and rank-2 proposals, showing that the EBM does understand this proposal’s limitations with a qualitative significance to the energy output, if not the reason.

As a second case study, we examine the target molecule in Fig 5 (bottom), published as the C–N coupling of an aryl iodide with a secondary alkyl amine. Interestingly, our EBM very slightly favors the C–N coupling with an aryl bromide over the aryl iodide. This may seem counterintuitive since the aryl C–I bond is weaker and iodides are generally better leaving groups. However, we cannot rule out that the aryl bromide is still reactive enough for such a C–N coupling reaction, and because aryl bromides tend to be cheaper than aryl iodides, the aryl bromide may be preferred by a chemist. That being said, the EBM’s assigned energies are essentially the same, with +1.104 for the aryl bromide verus +1.105 for the aryl iodide, which suggests they are perceived as equally valid options. In contrast, RetroSim proposed the aryl bromide as its rank-2 and the aryl iodide as its rank-13 suggestion, ranking the iodide much lower, which seems to indicate that our EBM has a relatively nuanced understanding of feasibility.

Lastly, we include several examples of “failure” cases in Additional file 1: Section S6 where the EBM worsened the rank of the published reaction relative to the original proposer. However, we also argue that even in such cases, EBM’s suggestions are plausible alternative strategies.

## Conclusion

Computer-aided retrosynthetic planning software can assist chemists by not just suggesting a pool of promising routes, but also ranking them in order of predicted plausibility. They can provide additional ideas for routes chemists may not have considered before, especially when synthesising novel molecules for which no literature precedent exists. Improving these data-driven retrosynthesis tools can benefit chemists in numerous applications of organic synthesis, ranging from small molecule drug discovery to material science.

Our work approaches the problem of retrosynthesis from the perspective of learning to re-rank the suggestions of existing one-step models to improve their performance. Through this work, we have designed and validated an energy-based approach to re-ranking, and explored 3 diverse architectures for the EBM. Firstly, we could significantly improve the performance of two template-based models: RetroSim and NeuralSym. We also boost GLN’s top-*N* accuracies across the board, with the largest improvements for $N\in \left\{ 3,5,10\right\}$. Improving template-based models is especially valuable as they can provide literature precedents responsible for each prediction, making them more chemically explainable than template-free models. Even within template-based models, RetroSim is extraordinarily transparent due to its parameter-free design; we know exactly which reaction precedent was used to generate each reactant-set proposal with a one-to-one correspondence. With re-ranking, we can enjoy this transparency with virtually the same top-1 accuracy as GLN which, although a template-based approach, is less interpretable in terms of how it exactly derives each proposal. While the top-1 accuracy of RetroXpert, a template-free approach, could not be improved, we still enhance its top-3 to top-50 accuracies. Finally, we show that re-ranking a combination of GLN and RetroSim can be superior than re-ranking just GLN or RetroSim.

Since the only input our EBM requires is a product with a pool of corresponding reactant-set proposals, our EBM works as a simple and convenient plug-and-play framework and can be applied to any one-step retrosynthesis method. Furthermore, the energy-based formulation allows for great flexibility in model design because the EBM only needs to output a single number; thus, there is great freedom to further enhance its architecture, for example, with extra chemical knowledge or meta-information about the one-step model. Pairing one-step proposal with energy-based re-ranking could also increase the chances of finding complete multi-step routes.

## Supplementary Information

**Additional file 1.** Additional details of molecular representations, datasets and preprocessing steps, hyperparameters for model experiments, additional re-ranking results and metrics, and example incorrect re-ranking predictions.

## Data Availability

All code for data preprocessing, proposer training, EBM training and evaluation is open-sourced at https://github.com/coleygroup/rxn-ebm. All data used in this study is freely available through the same link.
