# Supplementary material for: Improving the performance of models for one-step retrosynthesis through re-ranking
Source: J Cheminform. 2022 Mar 15;14:15. doi: 10.1186/s13321-022-00594-8 (PMC8922884; doi:10.1186/s13321-022-00594-8)
Supplement: Supplementary file 1 — Additional file 1. Additional details of molecular representations, datasets and preprocessing steps, hyperparameters for model experiments, additional re-ranking results and metrics, and example incorrect re-ranking predictions. [file 13321_2022_594_MOESM1_ESM.pdf]

Supporting Information: Improving the performance of models for one-step retrosynthesis through re-ranking

Min Htoo Lin,† Zhengkai Tu,‡ and Connor W. Coley*,¶

†*Division of Chemistry and Biological Chemistry, School of Physical and Mathematical Sciences, Nanyang Technological University, 637371, Singapore*

‡*Computational Science and Engineering, Massachusetts Institute of Technology, 77 Massachusetts Avenue, Cambridge, Massachusetts 02139, United States*

¶*Department of Chemical Engineering, Massachusetts Institute of Technology, 77 Massachusetts Avenue, Cambridge, Massachusetts 02139, United States*

E-mail: ccoley@mit.edu

Supporting Information Available

S1 Graph features used for the Graph-EBM

Table S1: Atom and bond features used for the Graph-EBM, adapted from ref. S1

Feature	Description	Feature Size
Atom type	Type of atom (e.g. C, N)	65
# Bonds	Number of bonds the atom has	10
Hybridization	sp, sp^2, sp^3, sp^3d or sp^3d^2	5
Formal charge	Integer electronic charge assigned to atom	5
Explicit valence	Explicit valence of the atom	7
# Hs	Number of bonded Hydrogen atoms	6
Chirality I	Unspecified, tetrahedral CW/CCW, or other	4
Chirality II	R, S or None	3
Aromaticity	Whether atom is part of an aromatic system	1
Bond type	Single, double, triple or aromatic	4
Bond stereochemistry	E, Z or None	3
Bond conjugation	Whether bond is conjugated	1
Bond aromaticity	Whether bond is part of an aromatic system	1

S2 Breakdown of 10 reaction types in USPTO-50K

Table S2: Breakdown of 10 reaction types across the entire USPTO-50K dataset[S2]

Reaction type	Reaction type name	Percentage (%)
1	Heteroatom alkylation and arylation	30.3
2	Acylation and related processes	23.8
3	C-C bond formation	11.3
4	Heterocycle formation	1.8
5	Protections	1.3
6	Deprotections	16.5
7	Reductions	9.2
8	Oxidations	1.6
9	Functional group interconversion (FGI)	3.7
10	Functional group addition (FGA)	0.5

S3 Extra steps to clean the USPTO-50K dataset

1. Remove reaction SMILES strings with products that are shorter than 3 alphabet characters and clearly incorrect: for example, organic reactants giving just 1 equivalent of water as the product. There were 4 such reactions in the training dataset.

2. We canonicalized all reactions using RDKit. Afterwards, duplicated reactions were removed such that only one unique copy of a reaction is present.

3. We were surprised to see that some reactions in the validation and test data that also appeared in the training data. This would exaggerate models' generalization performance. In total, 50 reactions in the test set and 44 in the validation set appeared in the training data, and were removed from the test and validation data. 6 test reactions that also appear in the validation data were also removed from the test set, so that the validation and test sets do not overlap.

S4 Details of model and training hyperparameters

The Adam optimizer[S3] with default hyperparameters was used to optimize the parameters of all EBM models. We experimented with multiple learning rate schedules such as the cyclic learning rate which has been previously shown[S4] to greatly speed up convergence. Ultimately however, we found that the most reliable and hyperparameter-insensitive choice is the PyTorch built-in ReduceLROnPlateau, which reduces the learning rate by a user-defined ratio whenever the top-1 accuracy on the validation data stops improving for a specified number of training epochs. To avoid overfitting, we used early stopping, which similarly monitors the validation top-1 accuracy and stops the training once it does not improve after a defined number of epochs. Specific hyperparameters are as follows:

S4.1 Feedforward-EBM

For the Feedforward-EBM, we use hidden sizes of $\{1024, 128\}$ for each of the 3 input networks, with one dropout layer (coefficient 0.2) in between. The final output layer has a dimension of 128 and is preceded by a dropout layer (coefficient 0.2). The PReLU activation is used throughout as well. The starting learning rate is set to 0.001, with a reduction factor of 0.4 and patience of 1 for ReduceLROnPlateau. During training, a batch size of 16 reactions is used while during evaluation a batch size of 32 is used. A patience of 3 is used for early stopping. The training takes about 2 to 3 hours on 1 RTX2080Ti GPU.

S4.2 Graph-EBM

For the Graph-EBM, we observed that slightly different hyperparameters are needed to achieve optimal performance to re-rank different one-step models. The differences lie mainly in whether a single shared MPNN encoder or two separate MPNN encoders are used to embed reactants and products, the pooling method to obtain the energy output given embeddings of reactants and products, the learning rate reduction factor for ReduceLROnPlateau, and early stopping patience.

First we describe the hyperparameters for re-ranking GLN, and then we specify the differences from this set for each of the other one-step models. We use the Gated Recurrent Unit (GRU) for the message passing operations. Following GraphRetro[S1] we use a message passing depth of 10 with dimension 300 for the GRU (ReLU activation). We additionally modify the GRU to have a dropout layer (coefficient 0.08) after each message passing iteration. Different from GraphRetro,[S1] we use two hidden layers instead of one for the final node update module $\mathbf{W}_o$, with dimensions $\{320, 300\}$ and a dropout layer (coefficient 0.08) in between. Also note that we use two MPNN encoder networks with identical architectures but separate parameters to encode product molecules separately from reactant molecules, which allows each MPNN to specialise to reactants or products, and resulted in a significant

performance boost. The pooled output embedding (300-dim) from each MPNN encoder is subjected to a small projection network (PReLU activation) of sizes $\{256, 200\}$ (dropout coefficient 0.12) and again we use a separate projection network for each MPNN encoder, to obtain the reactants embedding $\mathbf{R}_\mathcal{G}$ and product embedding $\mathbf{P}_\mathcal{G}$, which are concatenated with their difference $\mathbf{P}_\mathcal{G} - \mathbf{R}_\mathcal{G}$ and element-wise product $\mathbf{P}_\mathcal{G} * \mathbf{R}_\mathcal{G}$. Finally, the output network (PReLU activation) has dimensions $\{600, 300, 1\}$ with a dropout layer (coefficient 0.15) in between. The initial learning rate is set to 1e-4, with a learning rate reduction factor of 0.3 and patience of 1 for ReduceLROnPlateau. A patience of 3 is used for early stopping. A batch size of 2 reactions is used during training and 4 during evaluation, because these are the maximum we could fit on RTX2080Ti's. A GPU with even larger memory would allow larger batch sizes, which should speed up training. All gradients are clipped to 20 to stabilize training. Also note that we set a condition for training to stop if the learning rate falls below 1e-7, though in practice this was not triggered for re-ranking GLN.

For RetroSim, we observed that using just 1 shared MPNN encoder (depth = 10, dimension = 300, ReLU activation) for both reactants and products is sufficient, and using 2 MPNN encoders offers no conclusive improvement. We still use two separate projection networks, each of size $\{256, 200\}$, for reactants and products respectively after the shared MPNN encoder. However, unlike for GLN, we do not concatenate the reactants embedding $\mathbf{R}_\mathcal{G}$ and product embedding $\mathbf{P}_\mathcal{G}$. Instead, the energy is obtained directly through a dot product $\mathbf{R}_\mathcal{G} \cdot \mathbf{P}_\mathcal{G}$; thus, there is no output network (Fig S1). With significantly fewer parameters than using 2 separate MPNN encoders, we can afford a larger batch size of 4 during training and 8 during testing and accordingly, the initial learning rate is doubled to 2e-4, and we stop the training if the learning rate falls below 1e-6, which was triggered occasionally. Otherwise, we use the same hyperparameters as the 2 MPNN Graph-EBM used with GLN.

For NeuralSym, we also use just 1 shared MPNN encoder (depth = 10, dimension = 300,

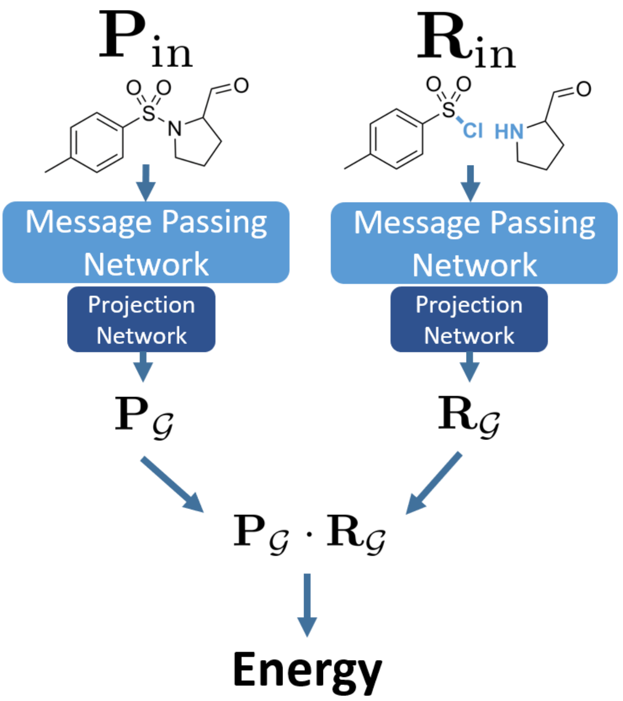

Figure S1: Schematics of Graph-EBM with a single shared MPNN encoder and dot product pooling

ReLU activation), with separate projection networks (ReLU activation) of size $\{256, 200\}$, followed by dot product $\mathbf{R}_{\mathcal{G}} \cdot \mathbf{P}_{\mathcal{G}}$ to get the energy. We found it helpful to use a more conservative learning rate reduction factor of 0.8, with the same patience of 1 for ReduceLROnPlateau, same early stopping patience of 3, and same learning rate floor of 1e-6.

With RetroXpert, due to the particularly poor re-ranking performance of the Graph-EBM, we explored more architectural and hyperparameter variations. While using just 1 shared MPNN encoder (depth = 10, dimension = 300, PReLU activation) proved optimal, we found it helpful to include a small pre-embedding layer $\mathbf{W}_{emb}$ of size $\{90\}$ in the MPNN encoder. Specifically, $\mathbf{W}_{emb}$ is applied on the initial message feature vectors before any message passing is done, and these transformed feature vectors are then fed into the GRU cell for the message passing iterations. Otherwise, we continue to employ two separate projection networks (ReLU activation) of size $\{256, 200\}$, followed by dot product $\mathbf{R}_{\mathcal{G}} \cdot \mathbf{P}_{\mathcal{G}}$ to get the

energy. The initial learning rate is set higher at 3e-4 with a learning rate reduction factor of 0.6, but otherwise the same patience of 1 for ReduceLROnPlateau and early stopping patience of 3.

Finally, for the union of GLN and RetroSim, we again use 1 shared MPNN encoder (depth = 10, dimension = 300, ReLU activation), as using two MPNN encoders would make training too slow, given that we have 100 training proposals instead of 50 (which roughly doubles training time). This is followed by separate projection networks (ReLU activation) each of size $\{256, 200\}$, and dot product pooling $\mathbf{R}_{\mathcal{G}} \cdot \mathbf{P}_{\mathcal{G}}$ to get the energy. A batch size of 2 reactions is used during training and 4 during evaluation, and accordingly, the initial learning rate is set to 0.0001 with a learning rate floor of 1e-7 (both same as for re-ranking GLN). Just as with RetroXpert, we found it beneficial to use a more conservative learning rate reduction factor of 0.8, with the same patience of 1 for ReduceLROnPlateau but a longer early stopping patience of 4.

The time taken for training the Graph-EBM depends on the proposer and exact hyperparameters used (especially the ReduceLROnPlateau patience, learning rate reduction factor, and early stopping patience). From our experience, training the Graph-EBM to re-rank RetroSim and NeuralSym can be as fast as 16 hours on 4 RTX2080Ti GPUs. For GLN, the training is slightly slower and takes about 27 hours on 4 RTX2080Ti GPUs. With RetroXpert, it takes about 20 hours on 4 RTX2080Ti GPUs. Lastly for the union of GLN and RetroSim, as we have 100 training proposals, double the 50 for re-ranking individual proposers, the training time is expected to take longer, and in practice about 46 hours on 4 RTX2080Ti GPUs. We observed a GPU scaling factor of roughly 1.5x, which means training the Graph-EBM to re-rank RetroSim should take about 24 hours on 2 RTX2080Ti GPUs, and about 36 hours on a single RTX2080Ti GPU.

S4.3 Transformer-EBM (TF-EBM)

4.3.1 Architecture

Other than the FF-EBM and Graph-EBM, we can also represent each reactant and product using SMILES, and encode each reaction with the Transformer[S5] (Fig S2). At the core of the Transformer, self-attention allows the Transformer to decide, for each token in the sequence, how much it should interact with every other token and itself. We refer readers to refs. S5 and S6 for comprehensive explanation of the Transformer. In retrosynthesis, self-attention could enable the model to capture complex interactions between atoms in a reaction. We use the atom-level tokenizer from ref. S7 to split each reaction SMILES into tokens, and apply the Transformer to obtain a vector for each input token. To summarise the reaction as one fixed-length vector, we use the classification ([CLS]) token pooling, which gathers context from all tokens through self-attention. Finally, the energy is obtained with a small feedforward network on this [CLS] pooled vector. We mainly based our implementation on the OpenNMT-py[S8] package.

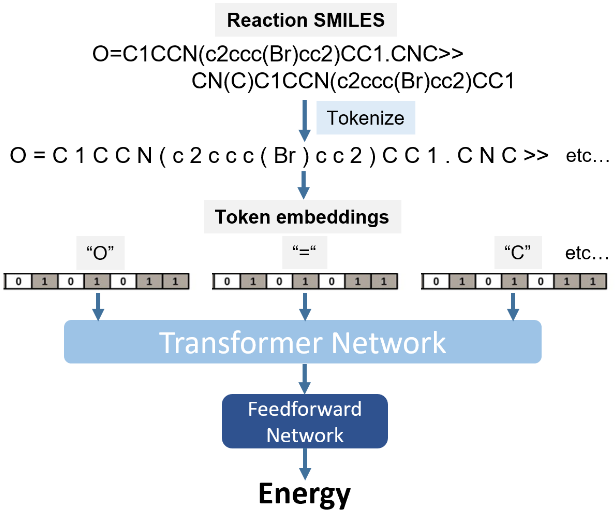

Figure S2: Schematics of the Transformer-EBM

4.3.2 Hyperparameters

For the Transformer encoder module in the Transformer-EBM, we use an embedding size of 256, with 3 layers of size 256. A coefficient of 0.05 is used for encoder dropout layers and 0.025 for the attention dropout. 4 attention heads and a filter size of 512 are used. The max sequence length is set to 256. [CLS] pooling is used to aggregate all processed token embeddings of the reaction SMILES into a single vector, on which an output network with sizes $\{128, 1\}$ is applied. The initial learning rate is set to 2e-3, with a learning rate reduction factor of 0.6, patience of 2 for ReduceLROnPlateau and early stopping patience of 5. A batch size of 8 is used both during training and evaluation. The training takes about 14 hours on 4 RTX2080Ti GPUs.

4.3.3 Results

Table S3: Results of re-ranking RetroSim on the USPTO-50K test dataset. Bolded values refer to the best top-N accuracy for that one-step model. We report the average of 3 experiments where both the proposer and re-ranker are initialized with a different random seed, with the standard deviation in parentheses.

Models	Top-N accuracy (%)					
	1	3	5	10	20	50
RetroSim	35.7 (±0)	53.3 (±0)	62.0 (±0)	73.4 (±0)	82.3 (±-)	88.5 (±-)
RetroSim + FF-EBM	49.7 (±0.34)	72.3 (±0.21)	79.4 (±0.15)	85.5 (±0.13)	88.1 (±0.07)	**88.9** (±0.01)
RetroSim + Graph-EBM	**51.8** (±0.43)	**74.5** (±0.37)	**81.1** (±0.17)	**86.4** (±0.13)	**88.5** (±0.02)	**88.9** (±0.00)
RetroSim + TF-EBM	23.2 (±0.66)	45.7 (±0.33)	57.3 (±0.13)	72.4 (±0.19)	83.4 (±0.27)	88.6 (±0.03)

We observed that the TF-EBM does poorly, failing to even recover RetroSim's original top-N accuracies. When we attempted to train the Transformer-EBM for an extended duration, the training top-1 accuracy consistently plateaued at about 33%. Due to its poor performance, we do not report the TF-EBM's results for the remaining one-step models. One hypothesis is that the TF-EBM struggles to learn, from reaction SMILES, the fine-grained chemical rules necessary to accurately distinguish chemically similar reactant-sets. Two chemically

similar reactant-sets can have very different SMILES due to RDKit canonicalization, which may exacerbate the learning difficulty in this re-ranking setting, which is different in nature from the original task of reactant-set generation.

We note that the lackluster performance of the TF-EBM is not in contradiction with the success of the Transformer architecture for sequence-to-sequence tasks in chemistry, including the task of one-step retrosynthesis itself. Sequence-to-sequence tasks typically use an autoregressive loss and model the task as conditional SMILES generation. This EBM formulation, on the other hand, is more similar to the Bidirectional Model used in S9, which also exhibits low performance, despite making use of bidirectional context similar to BERT.

S5 Additional metric to evaluate the EBMs' re-ranking performance

The primary objective of our work is to demonstrate that re-ranking with trained EBMs can improve the performance of existing one-step retrosynthesis models. Therefore, we believe that our reported metrics of top-N accuracy and Mean Reciprocal Rank (MRR) before re-ranking and that after re-ranking suitably highlights the benefits of our approach. Still, to substantiate our case further, we also report the area under the top-N curve as an additional metric.

S5.1 Area under the top-N curve (AUC-N)

The AUC-N depends on the choice of N, and we report the values for $N \in \{3, 5, 10, 20, 50\}$. The area was calculated by the sum of rectangles with one rectangle for each top-N accuracy, which is finally normalised by dividing by N, the total number of rectangles. Thus, if $N = 1$, the area simply equals the top-1 accuracy. The formula is summarised as follows:

$$\text{AUC-N} = \frac{1}{N}\sum_{i=1}^{N} a_i \tag{1}$$

where a_i is the top-i accuracy, and N is the maximum top-N accuracy that we consider.

For the AUC-N, the trends parallel exactly that of top-N accuracy, as expected. As shown in table S4, re-ranking with Graph-EBM achieves the best metric for RetroSim, NeuralSym and GLN, while for RetroXpert, FF-EBM is superior to Graph-EBM, but both are worse than raw RetroXpert when N is 3. As for re-ranking the union of RetroSim and GLN with Graph-EBM, we also see that it achieves better AUC-N than re-ranking RetroSim alone or GLN alone.

Table S4: Results of re-ranking the four one-step models, as well as the union of RetroSim and GLN, on the USPTO-50K test dataset. Bolded values refer to the best area under the top-N curve for that one-step model. We report the average of 3 experiments where both the proposer and re-ranker are initialized with a different random seed, with the standard deviation in parentheses.

Models	Area under the top-N curve				
	3	5	10	20	50
RetroSim	0.451 (±-)	0.511 (±-)	0.603 (±-)	0.697 (±-)	0.799 (±-)
RetroSim + FF-EBM	0.618 (±0.002)	0.680 (±0.002)	0.757 (±0.002)	0.815 (±0.001)	0.858 (±0.001)
RetroSim + Graph-EBM	**0.646** (±0.005)	**0.707** (±0.004)	**0.778** (±0.002)	**0.828** (±0.001)	**0.864** (±0.000)
NeuralSym	0.571 (±0.002)	0.631 (±0.002)	0.708 (±0.002)	0.773 (±0.002)	0.830 (±0.003)
NeuralSym + FF-EBM	0.624 (±0.004)	0.684 (±0.003)	0.756 (±0.003)	0.810 (±0.003)	0.848 (±0.003)
NeuralSym + Graph-EBM	**0.637** (±0.003)	**0.698** (±0.003)	**0.769** (±0.003)	**0.818** (±0.003)	**0.852** (±0.003)
RetroXpert	**0.540** (±0.004)	0.577 (±0.004)	0.624 (±0.004)	0.664 (±0.005)	0.708 (±0.003)
RetroXpert + FF-EBM	0.536 (±0.002)	**0.588** (±0.002)	**0.649** (±0.001)	**0.697** (±0.000)	**0.739** (±0.001)
RetroXpert + Graph-EBM	0.484 (±0.010)	0.548 (±0.010)	0.627 (±0.007)	0.688 (±0.004)	0.736 (±0.001)
GLN	0.603 (±0.003)	0.656 (±0.003)	0.731 (±0.002)	0.800 (±0.001)	0.869 (±0.001)
GLN + FF-EBM	0.623 (±0.004)	0.688 (±0.003)	0.768 (±0.002)	0.831 (±0.002)	0.886 (±0.001)
GLN + Graph-EBM	**0.650** (±0.002)	**0.713** (±0.002)	**0.787** (±0.001)	**0.845** (±0.001)	**0.893** (±0.001)
RetroSim + GLN + Graph-EBM	0.655 (±001)	0.719 (±0.001)	0.798 (±0.001)	0.859 (±0.001)	0.910 (±0.001)

S6 Examples of egregious re-ranking

While we have mainly highlighted the strengths of our EBM re-ranking approach, we also wish to highlight a few "failure" cases, where the EBMs have worsened the rank of the published reaction relative to the original proposer. However, as we will see, this is not necessarily "bad", as the EBM's top-ranked suggestions can still be useful.

In the first example (Fig S3), the published reaction is the formation of an ether linkage between a phenol and a primary alcohol. While RetroSim ranked this 4th, the Graph-EBM has worsened its rank to 7th. The Graph-EBM's top-1 proposal (ranked 11th by RetroSim) is a Heck coupling between a phenyl bromide and a terminal alkene. While there is no guarantee this reaction would work, it is not an absurd suggestion.

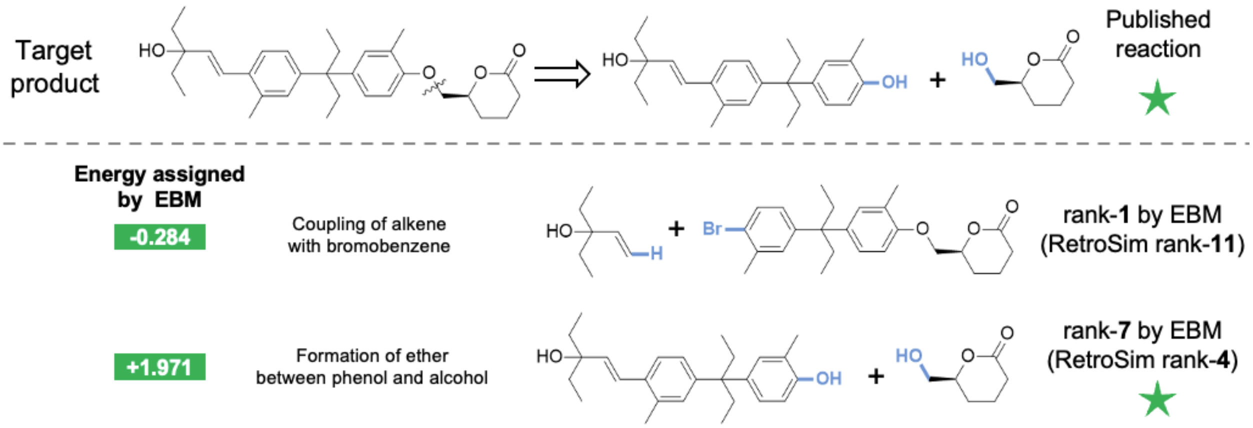

Figure S3: Graph-EBM prefers Heck coupling between a phenyl bromide and a terminal alkene, over etherification

Secondly, the published reaction as shown in Fig S4 is the oxidation of a secondary alcohol to a ketone. Such oxidation reactions seem rather straightforward, and was ranked 2nd by RetroSim, but pushed to 9th by the Graph-EBM. The top re-ranked suggestion (ranked 6th by RetroSim) is the Friedel-Crafts acylation of the benzene ring at the position para to the methoxy group. While this is certainly a valid chemical transformation, it may suffer from reduced selectivity due to the ortho-acylated side-product. Although the oxidation may be more selective, the EBM's top-1 proposal may still work in practice and may simply reflect

an overall bias in the dataset toward bimolecular reactions rather than unimolecular redox manipulations.

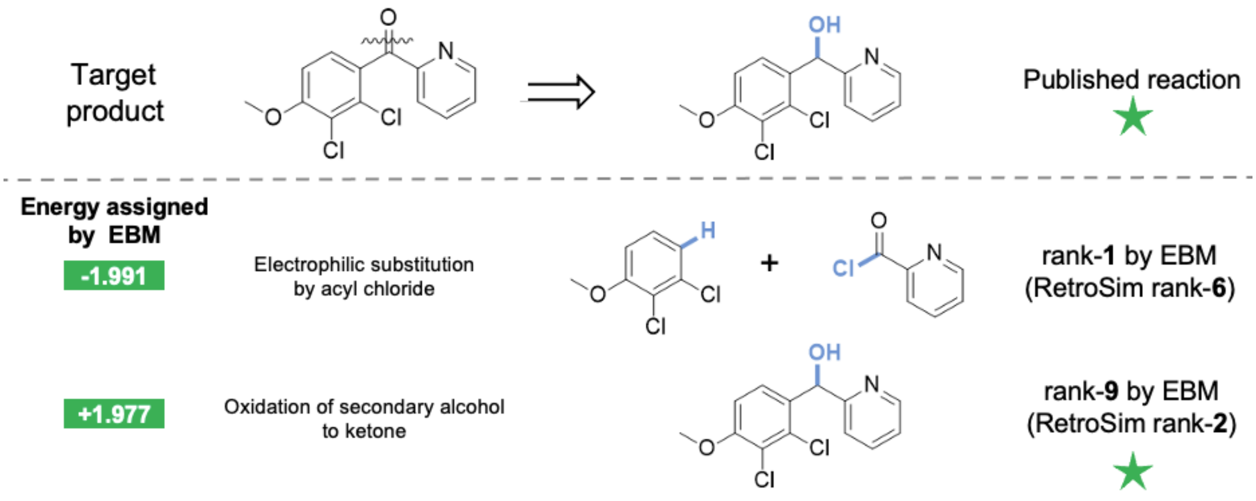

Figure S4: Graph-EBM favors electrophilic substitution over oxidation

Next in Fig S5, the published reaction involves a Grignard coupling between phenyl magnesium and a Weinreb amide; in fact, this is the second step of the named Weinreb ketone synthesis, which is a reliable method of selectively synthesising ketones without the alcohol side-product from over-addition of the organometallic reagent to the carbonyl center. The Weinreb amide is usually synthesised from the reaction of N,O-dimethylhydroxylamine with an acyl chloride precursor. While RetroSim ranked this 4th, the Graph-EBM has worsened its rank to 8. Instead, the Graph-EBM's rank-1 proposal is the coupling between a phenyl bromide and an acyl chloride to form the ketone. Presumably, the phenyl amine group acts as an electron-donating group to activate the ortho-bromo position towards electrophilic attack by the acyl chloride. Actually, this reaction seems practical too, and could yield the desired ketone. Again, this example highlights the subjectivity of evaluating reaction proposals, especially without considering other factors like reaction conditions.

The last example (Fig S6) involves the coupling between a nitrogen atom on a heterocycle (1h-Pyrazolo[3,4-d]pyrimidine) and iodobenzene to install the benzene ring onto the nitrogen. This published reaction was ranked 1 by RetroSim, but only 12th by the Graph-EBM. The

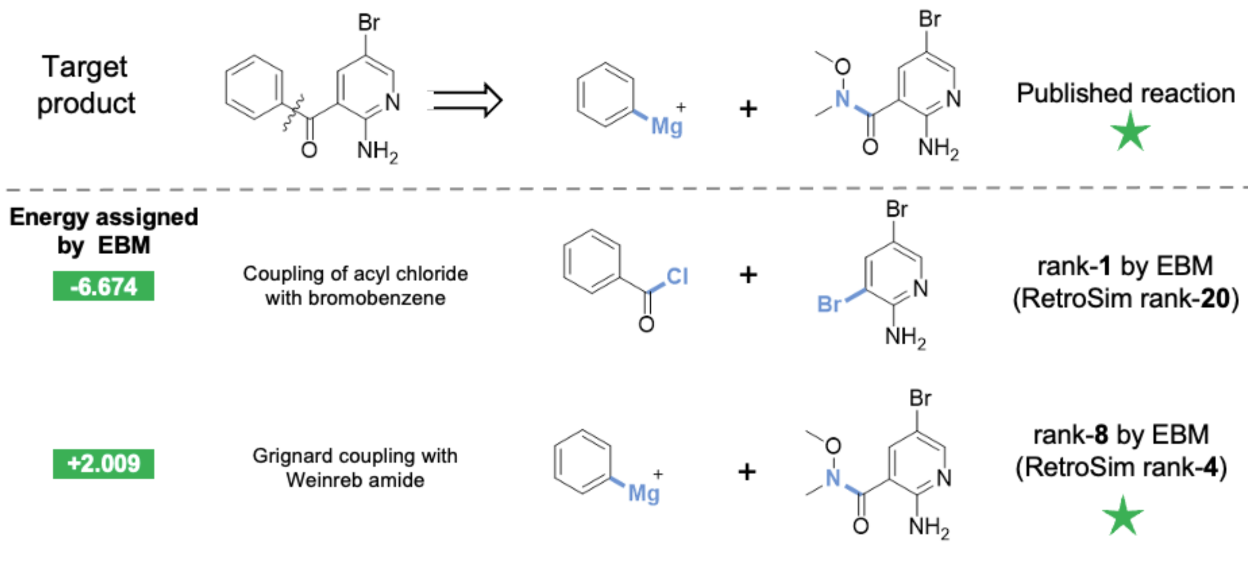

Figure S5: Graph-EBM favors the coupling of acyl chloride with bromobenzene over the Grignard coupling with a Weinreb amide

top proposal by the Graph-EBM is the substitution of a chlorine side group on the N-heterocycle with an amino group using ammonia. It is difficult to judge which reaction is more favorable in practice, which may boil down to the multi-step strategy being employed rather than the single-step chemical feasibility.

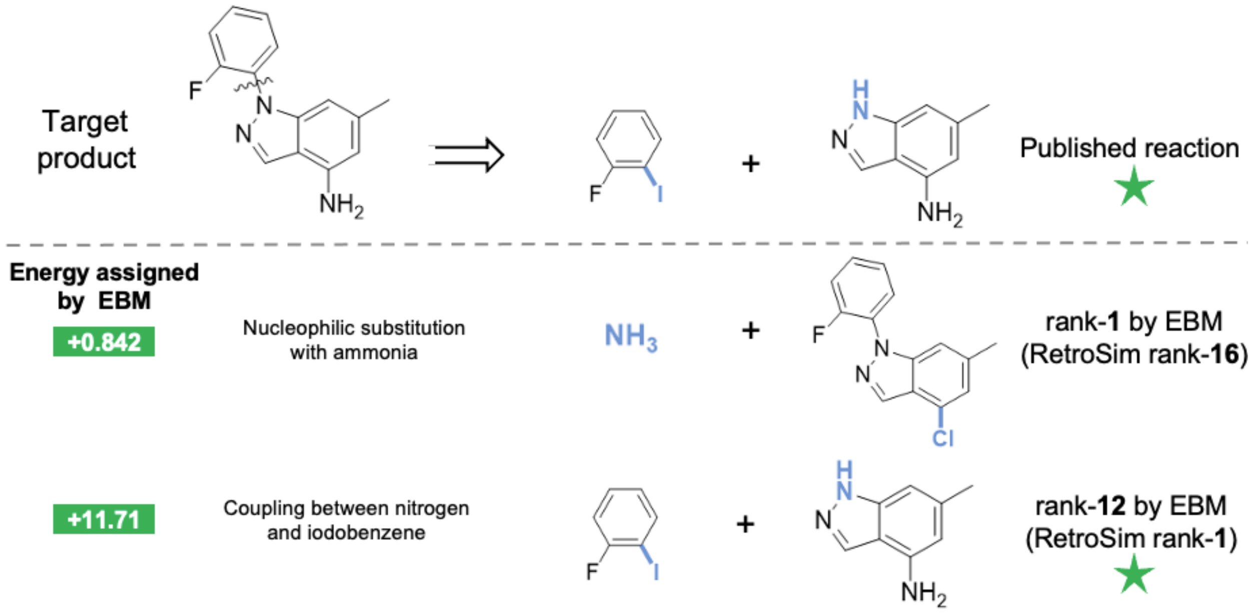

Figure S6: Graph-EBM favors the nucleophilic substitution of a heteroaryl chloride with ammonia over the coupling of a ring nitrogen and iodobenzene

References

(S1) Somnath, V. R.; Bunne, C.; Coley, C. W.; Krause, A.; Barzilay, R. Learning Graph Models for Template-Free Retrosynthesis. *arXiv preprint arXiv:2006.07038* **2020**,

(S2) Schneider, N.; Stiefl, N.; Landrum, G. A. What's what: The (nearly) definitive guide to reaction role assignment. *Journal of chemical information and modeling* **2016**, *56*, 2336–2346.

(S3) Kingma, D. P.; Ba, J. Adam: A method for stochastic optimization. *arXiv preprint arXiv:1412.6980* **2014**,

(S4) Smith, L. N.; Topin, N. Super-convergence: Very fast training of neural networks using large learning rates. Artificial Intelligence and Machine Learning for Multi-Domain Operations Applications. 2019; p 1100612.

(S5) Vaswani, A.; Shazeer, N.; Parmar, N.; Uszkoreit, J.; Jones, L.; Gomez, A. N.; Kaiser, L.; Polosukhin, I. Attention is all you need. *arXiv preprint arXiv:1706.03762* **2017**,

(S6) The Illustrated Transformer. `http://jalammar.github.io/illustrated-transformer/`, Accessed: 2021-05-15.

(S7) Schwaller, P.; Laino, T.; Gaudin, T.; Bolgar, P.; Hunter, C. A.; Bekas, C.; Lee, A. A. Molecular transformer: a model for uncertainty-calibrated chemical reaction prediction. *ACS central science* **2019**, *5*, 1572–1583.

(S8) Klein, G.; Kim, Y.; Deng, Y.; Senellart, J.; Rush, A. OpenNMT: Open-Source Toolkit for Neural Machine Translation. Proceedings of ACL 2017, System Demonstrations. Vancouver, Canada, 2017; pp 67–72.

(S9) Sun, R.; Dai, H.; Li, L.; Kearnes, S.; Dai, B. Energy-based View of Retrosynthesis. *arXiv preprint arXiv:2007.13437* **2020**,
